# Medical, epidemiologic, and social aspects of aging urinary incontinence questionnaire: Study protocol for the translation and validation of a Chinese language version

**DOI:** 10.1097/MD.0000000000017719

**Published:** 2019-11-01

**Authors:** Yuanjie Sun, Yan Liu, Tongsheng Su, Jingxue Yuan, Zhishun Liu

**Affiliations:** a Department of Acupuncture Guang’anmen Hospital, China Academy of Chinese Medical Sciences, Beijing, China; b Key Laboratory of Chinese Internal Medicine of Ministry of Education, Dongzhimen Hospital, Beijing University of Chinese Medicine, Beijing, China; c Shanxi Province Hospital of Traditional Chinese Medicine, Xi’an, China; d Beijing University of Chinese Medicine, Beijing, China.

**Keywords:** Medical, Epidemiologic, and Social Aspects of Aging questionnaire, mixed urinary incontinence, reliability, urgency-predominant mixed urinary incontinence, validity

## Abstract

**Introduction:**

Mixed urinary incontinence (MUI) is a coexistence of both urgency urinary incontinence and stress urinary incontinence. Medical, Epidemiologic, and Social aspects of Aging (MESA) questionnaire is a validated and commonly used tool to diagnose predominant components of it and assess the severity, which can offer help in clinic. However, MESA questionnaire is still not available in China. The aim of the study is to translate English MESA questionnaire into a Chinese version, adapt it in Chinese culture, and validate the measurement properties among female patients with MUI and urgency-predominant MUI.

**Methods:**

MESA questionnaire will be translated and culturally adapted in China. The validation will be embedded in a multicentered randomized controlled trial targeted at women with urgency-predominant MUI. Apart from MESA questionnaire, 3 groups of patients are to receive clinical extended assessment, keep 3-day voiding diary, and complete International Consultation on Incontinence Questionnaire Short Form to evaluate the measurement properties of reliability and validity (internal consistence, test-retest reliability, construct validity, and responsiveness).

**Discussion:**

If MESA questionnaire is of relatively high reliability and validity in diagnosing subtypes of MUI and assessing the severity, it can help to choose more appropriate therapy for patients and simplify the workload of clinicians.

**Trial registration:**

ClinicalTrials.gov NCT03803878, January 11, 2019.

## 1 Introduction

Mixed urinary incontinence (MUI) is defined as complaint or involuntary loss of urine associated with urgency and also with effort or physical exertion or on sneezing or coughing.^[1]^ It is regarded as urgency-predominant when urgency incontinence episodes dominate over stress ones and stress-predominant when stress incontinence episodes dominate.^[2]^ The treatment of MUI often begins with the most bothersome symptoms and the determination of predominant components can help to select optimal therapies for patients. At present, however, consensus is still lacking on the diagnostic criteria for MUI.^[2]^ Patient questionnaire, together with history and physical examination, voiding diary, urinalysis and urinary tract infection, post-void residual volume, urodynamics, pad testing, and imaging are important tools in the diagnosis and categorization of MUI recommended by 2018 EAU guideline.^[3]^

Medical, Epidemiologic, and Social Aspects of Aging (MESA) questionnaire is a reliable and validated tool developed and validated to identify the urgency- or stress-predominant component of MUI and assess the severity of symptoms. It is developed in MESA project, an observational study funded by the National Institutes on Aging (NIA) in 1983. In the project, 1955 seniors from a sample of 13,912 households in Michigan were interviewed and followed to investigate the epidemiology of urinary incontinence (UI).^[4]^

The MESA questionnaire is consisted of 2 separate parts, with 6 questions concerning urgency urinary incontinence (UUI) and 9 concerning stress urinary incontinence (SUI). The maximum total score of UUI is 18 and SUI is 27, with 0 to 3 allocated to the answers of never/rarely/sometimes/often. Urge/stress index is applied to detect urgency-/stress-predominant MUI. The indexes are obtained by dividing the actual score of each category by the maximum total possible. For example, the urge index is 50% with actual urge score of 9 dividing the maximum total urge score of 18, and the stress urge index is 33% with actual stress score of 9 dividing the maximum total stress score of 27. The MUI is categorized as urgency-predominant when urge index is greater than the stress index, and vice versa.^[5]^ To assess the severity of incontinence, the total score of urgency (18) and stress (27) are divided into third degrees. For urgency category, scores of 1 to 6 is assigned as mild, 7 to 12 moderate, and 13 to 18 severe. For stress category, scores of 1 to 9 is assigned as mild, 10 to 18 as moderate, and 13 to 18 as severe.^[5]^ The severity of UI can be measured either by total score of questionnaire or sub scores of urgency and stress.

MESA questionnaire is of relatively high reliability and validity. It can serve both as diagnosis tool and outcome assessment. To evaluate the test-retest reliability, questions were asked at the beginning and end of an interview. Agreement of late question to early question about incontinence reached 97.1%,^[6]^ and the agreement coefficient of incontinence frequency was 0.89.^[7]^ The UI reported by MESA questionnaire among women revealed 79.2% correspondence with clinician's assessment, in which a standardized history taking, physical examination, and simple provocative stress test are included.^[6,8]^ The type change and severity of incontinence over a 1- to 2-year period can also be detected by MESA questionnaire.^[7]^

MEAS questionnaire was validated in a large sample of population. In the process, researchers made great efforts to reduce underreport and avoid bias as far as possible.^[6]^

At present, MESA questionnaire is still inaccessible in China. The study aims to translate MESA questionnaire into Chinese, culturally adapt it in China and evaluate the reliability and validity of it, so that to choose a more appropriate therapy for female patients with predominant MUI and simplify the workload of clinicians.

## 2 Methods

### 2.1 Study design

The Chinese-version MESA questionnaire will be validated along with a multicentered randomized controlled trial (EASE-UMUI) targeted at women with urgency-predominant MUI. EASE-UMUI trial is approved by institutional review board of Guang’anmen Hospital, with number of 2018-163-KY. The protocol has been registered in ClinicalTrials.gov with number of NCT03787654 on December 22, 2018.

This validation study is also supported by institutional review board of Guang’anmen Hospital with number of 2018-162-KY. The protocol has also been registered in ClinicalTrials.gov with number of NCT03803878 on January 11, 2019.

Six hospitals around China will participate the validation, which are Guang’anmen Hospital, China Academy of Chinese Medical Sciences; Jiangsu Province Hospital of Traditional Chinese Medicine, Shanxi Province Hospital of Traditional Chinese Medicine, Guangdong Province Hospital of Traditional Chinese Medical, the First Hospital of Hunan University of Chinese Medicine, and Hengyang Hospital Affiliated to Hunan University of Chinese medicine.

**Figure 1:**
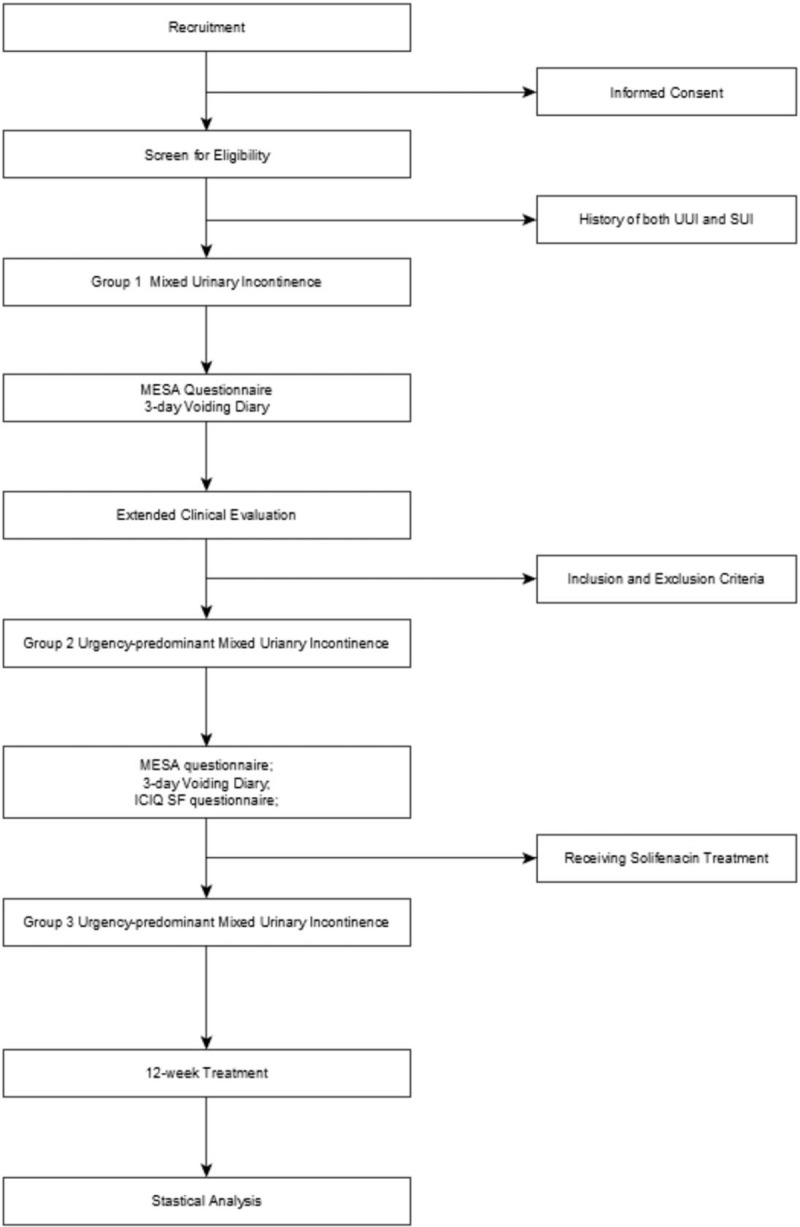
Study flowchart. ICIQ SF = International Consultation on Incontinence Questionnaire Short Form, MESA = Medical, Epidemiologic, and Social aspects of Aging, SUI = stress urinary incontinence, UUI = urgency urinary incontinence.

Data will be collected several times in the process of EASE-UMUI, and the observation and data collection will not interference the RCT trial (Fig. 1).

### 2.2 Recruitment and consent

Women will be recruited from both hospitals and communities by advertisements on newspaper, Web site, and posters. Eligible patients will be well informed of the situation of the trial and written informed consent will be signed before participating.

### 2.3 Population

The diagnosis function of MESA questionnaire will be validated among women with MUI, whereas the outcome assessment function of it will be validated among women with urgency-predominant MUI. Three subgroups of patients will be enrolled to assess the measurement properties.

1. Group 1 is anticipated to consist of 300 women with MUI. Patients visit any of the 6 hospitals and diagnosed with MUI by history taking will be invited.
2. Group 2 is anticipated to consist of 282 women with urgency-predominant MUI.
3. Group 3 is anticipated to consist of 94 women with urgency-predominant MUI.

Patients will be allowed to be participants of the 3 groups at the same time.

Female patients with medical history of both UUI and SUI will be enrolled into group 1. For patients in group 2 and 3, all the inclusion criteria and none of exclusion criteria are required to meet.

### 2.4 Inclusion criteria

Female patients diagnosed with MUI in accordance with EAU guideline by history taking, physical examination, laboratory tests, and specialist diagnosis.

1. Age between 18 and 80 years old;
2. At least 4 episodes of UUI in 3-day voiding diary;
3. UUI dominates >50% of the total incontinence episodes in 3-day voiding diary;
4. Positive cough test;
5. A voluntarily signed written informed content.

### 2.5 Exclusion criteria

1. Having pure SUI, pure UUI, overflow UI, or neurogenic bladder;
2. Uncontrolled urinary tract infection;
3. Tumor in urinary system or pelvic organs;
4. Pelvic organ prolapse ≥degree II;
5. Residual urine volume ≥100 mL;
6. Maximum flow rate <15 mL/s;
7. In the past 1 month, receiving treatment of acupuncture or positive medications targeted at incontinence, such as antimuscarinic drugs;
8. Underwent anti-incontinence or pelvic organ surgery, including metrectomy;
9. Complication of severe diabetes or hypertension;
10. Complication of diseases in nervous system that could hamper hypourethral function, such as multiple sclerosis, senile dementia, Parkinson disease, spinal cord injury, cauda equina nerve injury, and multiple system atrophy;
11. Severe complications in cardiac, lungs, cerebrum, hepar, renal system, psychonosology and coagulation function, or obvious cognitive disability;
12. Installed a cardiac pacemaker;
13. Allergic to Solifenacin or with contraindications to antimuscarinic drug, including urinary retention, gastrointestinal peristalsis paralysis, myasthenia gravis, ulcerative colitis, and angle-closure glaucoma;
14. Allergic to metal or intolerant to the stimulation of electroacupuncture;
15. Pregnant or plan to conceive in the future 1 year, or delivery in the past 1 year.

### 2.6 Outcome measures

The following measurement properties of MESA questionnaire will be tested in the study:

1. Internal consistency
2. Test-retest reliability
3. Construct validity
4. Responsiveness

### 2.7 Study process

The study consists of 2 parts, which are translation and adaptation of MESA questionnaire and validation. The project is permitted and supported by the questionnaire developer.

### 2.8 Translation and crosscultural adaption

The translation and crosscultural adaption of Chinese-version MESA were based on guidelines.^[9,10]^

#### 2.8.1 Translation

The MESA questionnaire was translated into 2 Chinese versions by 2 independent translators, who are Chinese native speakers, 1 with medical background and the other naive.

#### 2.8.2 Synthesis of the translation

Discussion of the 2 Chinese versions of MESA questionnaire was conducted to resolve any discrepancies and result in 1 refreshed version.

#### 2.8.3 Backtranslation

Since the MESA questionnaire is rather straightforward, a backtranslation was made by an English speaker without medical background and awareness of original version to elicit unexpected meanings.

#### 2.8.4 Expert Committee

To ensure cultural equivalence of Chinese and English versions, an expert committee consisting of urologist, methodologist, translators, language professionals, and researchers was initiated to discuss any discrepancies that may exist and develop a prefinal version.

#### 2.8.5 Test of the prefinal version

The prefinal Chinese version of MESA questionnaire is tested among 30^10^ persons with MUI to see whether the questions can be understood. If no ambiguities existed, there is not necessary to revise the questionnaire further, resulting in the final Chinese version MESA questionnaire.

### 2.9 Determining measurement properties of the MESA questionnaire

#### 2.9.1 Test-retest reliability

In screening period, patients in groups 2 are asked to complete MESA questionnaire at home. In about 1 to 2 weeks, those patients will refill the questionnaire in clinic. The time interval is set to erase of memory of answer before any symptom changes.

#### 2.9.2 Construct validity

Because MESA questionnaire can serve both as diagnosis criteria and outcome assessment, the construct validity will be evaluated among 2 groups of patients.

At the initial visit, patients will be asked 2 questions with the first one to ensure the occurrence of UI and the second to determine the existence of both SUI and UUI. Women with the history of MUI will be enrolled into group 1. Women in group 1 are asked to complete MESA questionnaire and 3-day voiding dairy at home. Later, those patients are recommended to receive extended clinical evaluation. Physical examination will be conducted, including cough stress test, urinalysis, and residual urine volume by ultrasonography. Individual information will be recorded including race, education, occupation, marriage, height, weight, the history of incontinence and other diseases, reproduction, menstruation, drinking, tobacco consumption, and a review of all medications and treatment. Physicians will make the final diagnosis of predominant MUI based on the information above and the records of 3-day voiding diary. The construct of MESA questionnaire in diagnosis will be validated by agreement with extended clinical assessment.

Since the score of MESA questionnaire can indicate the severity of UI, apart from 3-day voiding diary and MESA questionnaire, patients in group 2 are going to receive International Consultation on Incontinence Questionnaire Short Form (ICIQ SF) questionnaire. Three-day voiding diary is reliable and objective to measure the episodes of incontinence, indicating the severity of symptoms to some degree. ICIQ SF is a validated questionnaire to evaluate both the severity of incontinence symptoms in the past 4 weeks.^[11,12]^ It is consisted of 4 items, including frequency, leakage amount, impact on QoL, and the cause of UI. The total score of the ICIQ SF ranges from 0 to 21, with a higher score indicating worse symptoms and QoL. For patients who fill and refill MESA questionnaire, the second result will be used to test construct validity.

#### 2.9.3 Responsiveness

The responsiveness of MESA questionnaire will be tested among patients in group 3 with 12-week interval.

At screening stage and baseline period, patients are asked to fill in both MESA and ICIQ SF questionnaire, and keep 3-day voiding diary. After receiving 12-week treatment of Solifenacin, patients are asked to refill the questionnaires and keep diary to assess the symptom changes.

#### 2.9.4 Sample size calculation

The test-retest reliability and construct validity are meant to be evaluated among at least 50 people,^[13]^ whereas the internal consistency should be validated in at least 100 people, with 4 to 10 patients corresponding to each item of questionnaire.^[14]^

Because the validation study is embedded in a 3-armed randomized controlled trial, the sample size is set on an overall consideration of the actual situation.

Group 1 is anticipated to consist of at least 300 patients with MUI, group 2 is consisted of 282 patients, and group 3 is consisted of 94 patients receiving treatment of Solifenacin.

### 2.10 Statistical analysis

#### 2.10.1 Test-retest reliability

Reliability concerns the degree to which patients can be distinguished from each other, despite measurement error.^[13]^

Test-retest reliability will be evaluated by calculating the intraclass correlation coefficient (agreement) with a corresponding 95% confidence interval. Reliability will be given a positive rating when the intraclass correlation coefficient is at least 0.70 in a sample size of at least 50 patients.^[13]^

#### 2.10.2 Internal consistency

Internal consistency is to reveal the extent to which items in a questionnaire or a (sub)scale are correlated to measure the same concept.^[13]^ Cronbach alpha is used to measure the correlation between the 6 items of urgency incontinence and 9 items of stress incontinence. Measurement properties of MESA questionnaire is regarded as sufficient if the value of Cronbach alpha is between 0.70 and 0.95. The data at week 12 will be analyzed since patients recover to different degree, thus bearing variability.

#### 2.10.3 Construct validation

Validity is the degree to which a questionnaire measures the construct it is supposed to measure.^[13]^ As there was no criterion standard in the current study, the validity of the MESA will be expressed in terms of the construct validity. Construct validity represents the extent to which scores on a specific questionnaire relate to other measures in a way that is in agreement with prior theoretically derived hypotheses concerning the concepts that are being measured.^[13]^ Construct validity of MESA was assessed by determining the correlation of the MESA with extended clinical assessment, 3-day voiding diary, and ICIQ SF. Spearman *r* (rank correlation) coefficients (nonparametric correlation) will be correlations, respectively. Strengths of correlation was categorized as high (*r* > 0.6), moderate (0.3 < *r* < 0.6), or low (*r* < 0.3).^[15]^ Construct validity was considered sufficient if at least 75% of the results were in line with the predefined hypotheses in a (sub)sample of at least 50 patients.^[13]^

#### 2.10.4 Responsiveness

Responsiveness is defined as the ability of a questionnaire to detect clinically important changes over time, even if these changes are small.^[13]^ Patients in group 3 will complete all questionnaires twice, with 12-week interval. The effect size and standardized response mean (SRM) of MESA will serve as measures of change. The effect size will be calculated by dividing the mean change in score between the 2 time points by the SD of the first measurement. The SRM will be calculated by dividing the mean change in score between 2 time points by the SD of this change. In accordance with Cohen: SRM of 0.2 to 0.4 is regarded as a small effect, 0.5 to 0.7 moderate, and 0.8 or higher large. Change scores of MESA questionnaire are expected to have a moderate correlation with changes in the incontinence episode and scores of ICIQ SF.

#### 2.10.5 Data management

The data will be recorded in CRF before put into Electronic Data Capture System (Linkermed Technology Co. Ltd.). The final database will be protected by password, available only to principal investigator and locked once finished. Both the CRF and dataset will be preserved for 5 years. Data from the trial will be shared by request to the corresponding author anonymously except for private information of patients.

## 3 Discussion

MESA questionnaire is a validated and user-friendly questionnaire to determine predominant components of MUI and assess the severity of UI in an office setting.^[5]^

The validation of Chinese language version MESA questionnaire is embedded in a 3-armed random controlled trial, with a sample of 282, to evaluate the effect of electroacupuncture VS Solifenacin on women suffering from urgency-predominant MUI. At enrolment period, 300 female patients with MUI are anticipated to be enrolled to validity its function of diagnosis. The test-retest reliability is tested in screening stage and baseline period with 1- to 2-week interval to avoid symptom changes, the agreement with 3-day voiding diary and ICIQ SF will be conducted among 282 women with urgency predominant. Responsiveness is validated among a subgroup of 84 patients before and after Solifenacin treatment for 12 weeks. For this reason, diagnosis function of MESA is validated among women with MUI, whereas the reliability and validity of MESA as outcome assessment will be tested in women with urgency-predominant MUI. Therefore, further study is needed to explore the validity in other types of UI, such as pure SUI, pure UUI, or stress-predominant MUI when necessary.

In MESA questionnaire, both total score and subscores of urge and stress can indicate the severity of incontinence. In the present study, the construct validity is tested by the total score with ICIQ SF and incontinence episodes recorded in 3-day voiding diary. In the future, the subscores of urge and stress might also needed to be validated in assessing the severity of predominant MUI separately.

If MESA questionnaire is proved to be of equal reliability and validity with extended clinical evaluation in diagnosing predominant components of MUI, optimal therapy can be selected to patients and the workload of clinicians can be relived to a great degree.

## Acknowledgments

Deepest appreciation to the consultant of the study—Ananias C. Diokno, member of Medical, Epidemiologic, and Social aspects of Aging (MESA) project, for his consistent support and guidance in the process of validation. His enthusiasm to medicine, modest characteristic, and care for health of people really touches and motivates me. To every member in Linkermed Technology Co. Ltd. and every researcher in the 6 center hospitals for their good advice and hard work.

## Author contributions

ZSL and YL conceived the idea of the trial. ZSL, YL, and YJS designed the study. YL is responsible for statistical analysis. This manuscript was drafted by YJS and revised by ZSL, TSS, and JXY. All authors read and approved the final manuscript.

Zhishun Liu orcid: 0000-0001-7570-8917.
